# Adolescents’ knowledge and beliefs regarding health risks of soda and diet soda consumption

**DOI:** 10.1017/S1368980022001719

**Published:** 2022-11

**Authors:** Caroline Miller, Joanne Dono, Maree Scully, Belinda Morley, Kerry Ettridge

**Affiliations:** 1 The University of Adelaide, School of Public Health, Adelaide, Australia; 2 Health Policy Centre, South Australian Health and Medical Research Institute, North Terrace, Adelaide 5000, Australia; 3 Cancer Council Victoria, Melbourne, Australia; 4 The University of Adelaide, School of Psychology, Adelaide, Australia

**Keywords:** Adolescent, Knowledge, Sugar-sweetened beverage, Soda, Diet soda, Health risks

## Abstract

**Objective:**

To examine Australian adolescents’ knowledge and beliefs regarding potential health consequences of soda and diet soda consumption and nutritional aspects of soda and explore associations with consumption.

**Design:**

A survey utilising a nationally representative sample (stratified two-stage probability design) assessed knowledge of nutritional contents and health consequences of soda, and beliefs regarding health risks of diet soda, and soda and diet drink consumption.

**Setting:**

Australia.

**Participants:**

9102 Australian school students (12–17 years) surveyed in 2018.

**Results:**

Adolescents had lower nutritional knowledge (sugar content (22·2 %), exercise equivalent (33·9 %), calories/kJ (3·1 %)) than general knowledge of health risks (87·4 %) and some health effects (71·7–75·6 % for tooth decay, weight gain and diabetes), with lower knowledge of heart disease (56·0 %) and cancer (19·3 %). Beliefs regarding health effects of diet soda were similar, albeit not as high. In general, female sex, older age and less disadvantage were associated with reporting health effects of soda and diet soda, and nutritional knowledge of soda (*P* < 0·001). Those reporting tooth decay, weight gain, heart disease and diabetes as health effects of soda and diet soda were lower consumers of soda and diet drinks (*P* < 0·001), as were those with higher nutritional knowledge (sugar content and exercise equivalent; *P* < 0·001).

**Conclusions:**

This study highlights possible knowledge gaps regarding the health effects of soda and nutritional knowledge for public health intervention. When implementing such interventions, it is important to monitor the extent to which adolescents may consider diet drinks as an alternative beverage given varied beliefs about health consequences and evolving evidence.

It is well known that consumption of sugar-sweetened beverages (SSB) is associated with numerous health risks, including obesity, dental caries, diabetes and cardiovascular risk factors^(1–4)^. SSB consumption among the general population is concerning; however, consumption among adolescents has been identified as particularly problematic globally^(5)^. This is due to adolescents’ high consumption levels^(6–8)^, and the fact that they are within a period where behaviours can become embedded and have long-term impacts on health status^(6,9)^. Soda is the most commonly consumed SSB among Australians and is the biggest contributor to sugar intake for adolescent males in Australia – the highest SSB consumers^(6,10)^.

Reducing consumption of SSB is a public health priority. While water is the ideal beverage to replace SSB, a variety of alternative beverages exist and there is risk that consumers may switch to artificially sweetened beverages (ASB) in reaction to health policy and interventions aimed at reducing SSB consumption^(11)^. This is concerning given evidence regarding the safety of artificial sweeteners is, at best, inconclusive, with conflicting results regarding health risks associated with consumption of artificial sweeteners^(12)^. A precautionary approach is warranted with respect to consumption of ASB so as not to inadvertently encourage consumption with SSB policies and interventions.

The determinants of health behaviour are complex, as evidenced by the multitude of health behaviour theories that offer frameworks to understand the social, environment and individual drivers of behaviour change^(13)^. Many of these theories acknowledge adequate knowledge of health risks and consequences of behaviours as antecedents to behavioural intentions and behaviour change. Public health interventions often target knowledge and perceptions of a health behaviour and associated health risks, as part of a multi-faceted approach to encourage behaviour change^(14–16)^. There is increasing evidence of the effectiveness of campaigns in reducing SSB consumption that target knowledge and educate consumers on health effects^(17)^. Policy makers require insight into the limitations and gaps in population knowledge, and hence the need for interventions. This would provide insight into the specific components of knowledge that may be best targeted to fulfil knowledge gaps, as well as specific groups who may benefit most from intervention.

While consumers, including adolescents, have a right to be informed of nutritional aspects of food and beverages they consume, many have low knowledge of the health effects of SSB consumption and find interpretation of nutrition information challenging^(18)^. This limited knowledge may contribute to unhealthy choices and increase health risks, e.g. weight gain or obesity. Adolescents themselves have been found to rate knowledge of health effects of SSB as an important resource for reducing consumption^(19,20)^. A qualitative study found young school children perceived improving knowledge as important in changing levels of SSB consumption^(21)^. Moreover, nutritional/health knowledge has been associated with better health behaviours in other contexts, such as increased vegetable consumption among school aged children^(14,22)^, good eating habits among adolescents^(23)^, and reduced unhealthy food consumption among children and young adolescents^(24)^. Despite this, the small number of studies that have specifically examined the association between knowledge and SSB consumption among adolescent samples have yielded inconsistent results. For example, two studies found higher nutritional knowledge was significantly associated with lower SSB consumption^(24,25)^. In contrast, knowledge was not found to be significantly associated with SSB consumption in two studies after adjustment for adolescent and parent demographics^(26,27)^. The inconsistency in results across these studies is similar to that found among adult samples; where few studies show evidence of association after adjusting for the effects of demographic characteristics^(15,28–31)^.

Adolescents’ knowledge and beliefs regarding SSB and ASB in terms of health and health risks have not been comprehensively assessed, with a notable absence of studies in the Australian context. Internationally, most studies regarding knowledge and understanding of the health risks associated with SSB consumption and nutritional knowledge have been conducted on adult populations^(15,25–28,30–33)^. Heterogeneity across the small number of adolescent studies in definitions, assessments and methods utilised has led to inconsistent results. Some studies indicate overall moderate-high levels of knowledge are found when general knowledge of health risk is assessed, for example, knowledge of health effects associated with consumption (e.g. diabetes) or overall nutritional knowledge^(25,26)^. More modest levels are found when more specific aspects of knowledge are assessed, for example, high sugar content in SSB, and energy expenditure to eliminate sugar consumed from SSB^(15,28,30–33)^. Factors such as being older, coming from households with greater incomes, education and non-hispanic have also been found to be associated with knowledge of health effects (weight gain, dental caries) and/or overall nutritional knowledge among two adolescent samples^(25,26)^. While, overall, consumers (adults and children) consider diet soda to be among the unhealthiest options when choosing from non-alcoholic beverages^(21,31,34–37)^, studies on children’s perceptions and beliefs regarding ASB are sparse^(21,36)^, and have not specifically assessed adolescents’ beliefs regarding health risks of consumption. As noted, consumers may switch to ASB to avoid consuming SSB in reaction to health policy aimed at reducing consumption of SSB^(11)^.

There is an absence of studies in Australia that have comprehensively investigated knowledge and beliefs regarding the health risks associated with SSB and ASB consumption among adolescents, including exploring how knowledge and beliefs may vary with consumption and demographic characteristics. Identifying demographic subgroups with lower knowledge can assist in identifying those who will benefit the most from intervention. Notably, we were also unable to find any studies with adolescent samples that explored how beliefs regarding health risks of consuming diet sodas may vary by demographic characteristics, and/or how level of diet drink consumption may vary with such beliefs.

The aim of this study was to investigate knowledge and beliefs associated with SSB and ASB consumption in the Australian adolescent population using data from the 2018 National Secondary Students’ Diet and Activity (NaSSDA) survey, a nationally representative survey of Australian secondary students. The present study focused on knowledge of health effects of soda consumption, and beliefs regarding health risks of diet soda consumption. We refer to beliefs rather than knowledge in relation to diet soda due to the evolving nature of this evidence. Specifically, we sought to determine among Australian adolescents: (i) the level of knowledge of general health risks and nutritional knowledge of soda consumption, and beliefs regarding the health risks associated with diet soda; and (ii) the degree to which level of health knowledge of soda and beliefs regarding diet soda are associated with socio-demographic characteristics, specifically, sex, socio-economic status and age. These demographic characteristics were selected based on evidence of variation in knowledge in previous studies of adolescents and adults^(25,26,30)^. We also explored associations between knowledge and beliefs and consumption of soda and diet drinks.

## Methods

### Design

The 2018 NaSSDA survey, a nationally representative survey of students in secondary school in Australia (year levels 8 to 11), was the sample source for this study. This survey employs a two-stage probability design with random selection of schools (first stage), and classes within schools (second stage), and stratified by the three education sectors (government, Catholic, independent) within each Australian state or territory. The NaSSDA survey collects data on Australian secondary students’ physical activity and eating behaviours. In 2018, we commissioned a series of additional questions in the NaSSDA survey to assess adolescents’ knowledge of the health risks of soda consumption and beliefs regarding diet soda consumption.

### Procedure

Students were asked to complete a web-based survey during a class period. Data were available from 9102 adolescents, yielding a student response rate of 67 %.

### Measures

#### Knowledge of general health risks from regular soda consumption

Based on questions used previously in similar studies^(30,38)^ adolescents were asked to rate the likelihood of future health problems if they consumed a 600 ml (20 fl oz) soft drink (soda) every day, with available responses of: *very likely, somewhat likely, neither likely nor unlikely, somewhat unlikely, very unlikely.* This quantity (600 ml) was selected in this study and previous studies with Australian samples as it is a common size of ready-to-consume refrigerated beverages in Australia and adolescents would therefore be familiar with this size^(30,38)^.

#### Knowledge of health effects of soda and beliefs regarding health effects of diet soda

Knowledge of health effects of soda consumption, and beliefs regarding health effects of diet soda consumption, were assessed with questions used in a number of other studies^(26,30,38–40)^. Adolescents were asked ‘Which of the following diseases or health effects would you associate with drinking soft drinks (soda)/diet soft drinks (soda)?’. Adolescents were provided with a list of options which included: diabetes, weight gain/obesity, heart disease, tooth decay, cancer, other illnesses, no illness or ‘unsure’.

To assess beliefs regarding relative healthiness of diet sodas compared to regular soda, adolescents were asked to rate whether they perceived diet soft drinks [sodas] as more or less healthy than regular soft drinks (or the same). This question was based on similar questions used in studies of Australian adults^(30,39)^.

#### Nutritional knowledge of soda

Three questions assessed adolescents’ knowledge of the nutritional content of soda. Adolescents were asked to indicate, with a 600 ml (20 fl oz) soft drink (soda) in mind, how many teaspoons of sugar were in the beverage, the total calories or kJ (converted to calories for analysis), and the minutes of jogging required to work off the beverage. Responses were categorised into correct ranges based on the contents of popular sodas, allowing variation in estimates of approximately 4–5 teaspoons, ∼50 calories or 15 min (specific ranges are available from results tables). A 600 ml beverage was chosen as the reference point for this question for the same reasons it was a referred to in the assessment of ‘knowledge of general health risks’ in this study, i.e. previous use in other Australian studies, common size for ready consumption in Australia, and adolescents’ familiarity with this size^(30,38)^.

#### Consumption of soda and diet drinks

Consumption was assessed with questions of the main NaSSDA survey that are kept consistent across survey years to enable monitoring of health behaviour^(41)^. Specifically, adolescents were asked to indicate how many cups (250 ml/∼9 fl oz) of soft drink (soda) they usually consumed either per day, week or month. Consumption of diet drinks (soft drinks (soda), sports drinks, and cordials) was assessed using the same format. Volumes were then categorised into low consumption (3 cups or less/week), and high consumption (4 cups or more/week) based on limits set in NaSSDA and other consumption studies^(41)^.

#### Demographics

Demographic information was collected regarding adolescents’ sex, age, school year level and main language spoken at home. Given that most adolescents who attend secondary school grades 8 to 11 in Australia are aged between 13 and 16 years, adolescents aged 12 years (0·4 % of the sample) were included with the 13-year-old age group, and adolescents aged 17 years (6·9 %) were included with the 16-year-old age group for the purpose of reporting and analyses. Adolescents were also asked to indicate their postcode to enable the determination of level of disadvantage and remoteness of their residence, based on Socio-Economic Index for Areas (SEIFA)^(42)^ and Australian Statistical Geography Standard Remoteness structure, respectively^(43)^. The SEIFA categorises areas based on a range of socio-economic factors such as employment status, income and household status. Scores were also categorised into tertiles that ranked areas from the most disadvantaged (low score) to least disadvantaged (high score). The Australian Statistical Geography Standard Remoteness structure categorises areas into ‘metropolitan’ to ‘very remote’ based on relative access to services, with categories: ‘major cities,’ ‘inner regional’ and ‘outer regional, remote or very remote.’ Due to the low proportion of adolescents (3·6 %) who resided in remote locations in this survey year, we combined regional and remote categories for reporting, consistent with reporting from an earlier NaSSDA survey^(41)^.

### Statistical analyses

All analyses were conducted using Stata Statistical software release 15^(44)^. Bivariate associations between knowledge or beliefs and demographics were tested using chi-square, and associations between knowledge or beliefs and consumption variables (regular soda and diet drink consumption; high *v*. low consumers for each beverage) were tested via a series of multilevel logistic regression models with random effect for school to adjust standard errors for correlations within and between schools. Models also adjusted for school type, state/territory, sex, age and level of disadvantage. OR were reported to describe the odds of being a high soda or diet drink consumer according to knowledge and beliefs regarding these beverages. The primary interest was the associations between knowledge (soda) or beliefs (diet soda) regarding, the health effects of a beverage and consumption of the same type of beverage. However, for completeness we also looked at associations between knowledge of the health effects of consuming soda with diet drink consumption, and vice-a-versa. The number of analyses undertaken and the large data set inflated the risk of Type I error, as such, we employed a conservative probability level (*P* < 0·001)^(45,46)^.

## Results

Adolescent characteristics are available from Table 1. There were slightly more females (52·1 %) than males (47·9 %) and adolescents from each of the older age groups (27·7 %–29·1 %) than those in the youngest age group (15·2 %) in the study sample. Most adolescents spoke predominantly English at home (86·1 %) and were from metropolitan areas (65·3 %). The sample consisted of more adolescents from areas of least disadvantage (40·5 %) than that of mid and most disadvantage (33·9 % and 25·6 %, respectively).

**Table 1:** Characteristics of adolescents

Characteristics (*n* 9102)	%
Age	
12–13 years*	15·2
14 years	29·1
15 years	27·7
16–17 years†	28·0
Sex	
Male	47·9
Female	52·1
Level of disadvantage (tertiles)	
Most disadvantage	25·6
Mid disadvantage	33·9
Least disadvantage	40·5
Remoteness	
Metro	65·3
Regional/remote‡	34·7
Main language spoken at home	
English	86·1
Other	13·9
Soda consumption	
None	21·5
Less than 1 cup/week	46·2
1–3 cups/week	19·1
4–6 cups/week	4·5
1 or more/d	8·8
Diet drink consumption§	
None	59·8
Less than 1 cup/week	26·3
1–3 cups/week	7·4
4–6 cups/week	2·2
1 or more/d	4·2

* Only 0.4 % of adolescents were aged 12 years; therefore, 12 and 13 year olds were reported in the same age category.

† Only 6.7 % of adolescents were aged 17 years; therefore, 16 and 17 year olds were reported in the same age category.

‡ Only 3.6 % were classified as remote; therefore, we combined remote and regional for simplicity in reporting.

§ Diet varieties of soft drink (soda), flavoured mineral waters, energy drinks, sports drinks and cordial.

### Knowledge and beliefs regarding health risks

Knowledge (agreement) that consuming a 600 ml bottle of soda every day would likely lead to health problems in the future was high overall at 87·4 % (see Table 2). However, knowledge of specific health effects or diseases associated with soda consumption was varied. High proportions of adolescents (70 %+) indicated that tooth decay, weight gain/obesity and diabetes were associated with soda consumption, 56·0 % indicated heart disease, and 19·3 % indicated cancer. Beliefs regarding specific health effects or illnesses associated with diet soda consumption reflected similar patterns to those found with soda and knowledge of health effects, albeit proportions were slightly lower, and 30·6 % reported being unsure, whereas only 12·1 % were unsure of health effects of soda consumption.

**Table 2:** Variation in knowledge and beliefs according to demographic characteristics

Knowledge (soda) or belief (diet soda)		Sex		*χ*^2^ *P*-value	Age (years)				*χ*^2^ *P*-value	Level of disadvantage			*χ*^2^ *P*-value
	Overall	Male	Female		12–13 years	14 years	15 years	16–17 years		Most	Mid	Least	
	%	%	%		%	%	%	%		%	%	%	
Likelihood would have health problems from daily 600 ml soda consumption													
Very/somewhat likely	87·4	86·7	88·0	0·056	87·2	87·4	87·1	87·8	0·879	85·7	87·2	88·6	0·006
Did not say likely	12·6	13·3	12·0		12·8	12·6	12·9	12·2		14·3	12·8	11·4	
Illnesses or health effects associated with consumption (prompted)													
Soda													
Tooth decay	75·6	70·2	80·5	<0·001	70·2	74·2	77·2	78·6	<0·001	66·2	76·4	80·8	<0·001
Weight gain/obesity	71·7	65·7	77·1	<0·001	66·8	70·0	73·2	74·6	<0·001	62·1	72·0	77·3	<0·001
Diabetes	72·7	68·5	76·5	<0·001	68·1	71·2	74·0	75·5	<0·001	63·2	73·3	78·1	<0·001
Heart disease	56·0	53·9	58·0	<0·001	47·0	53·7	58·7	60·8	<0·001	46·8	56·8	61·0	<0·001
Cancer	19·3	18·4	20·1	0·044	19·9	19·5	18·4	19·7	0·590	14·9	19·2	22·1	<0·001
Unsure	12·1	13·6	10·8	<0·001	15·8	13·3	11·6	9·5	<0·001	15·4	11·7	10·4	<0·001
None	5·6	8·3	3·2	<0·001	5·7	5·9	5·1	5·7	0·701	10·4	5·2	3·0	<0·001
Diet soda													
Tooth decay	55·9	52·4	59·0	<0·001	50·1	54·9	54·9	61·0	<0·001	46·0	56·2	61·6	<0·001
Weight gain/obesity	46·2	42·2	49·9	<0·001	39·6	45·0	46·0	51·4	<0·001	37·7	45·0	52·4	<0·001
Diabetes	46·0	42·4	49·2	<0·001	38·8	44·1	46·9	51·1	<0·001	38·1	45·6	51·2	<0·001
Heart disease	39·5	38·2	40·7	0·017	31·3	37·8	40·6	44·7	<0·001	31·5	39·5	44·4	<0·001
Cancer	16·8	16·4	17·2	0·323	14·5	15·6	16·7	19·3	<0·001	12·6	17·3	18·9	<0·001
Unsure	30·6	30·1	31·0	0·390	36·0	32·5	30·5	25·6	<0·001	35·2	30·4	27·9	<0·001
None	6·6	9·6	4·0	<0·001	7·0	6·4	6·7	6·4	0·861	11·7	6·3	3·7	<0·001
Relative healthiness of drinks: compared to soft drink (soda) diet soft drinks (sodas) are…													
More healthy	20·3	22·4	18·4	<0·001	23·9	21·5	19·0	18·3	0·001	20·1	20·8	20·0	<0·001
Less healthy	15·5	17·1	14·0		15·3	15·4	15·9	15·3		17·6	16·6	13·3	
The same	64·3	60·5	67·6		60·8	63·2	65·1	66·5		62·3	62·7	66·8	
In reference to a 600 ml bottle of soda, knowledge of…													
Teaspoons of sugar													
Approx. correct (11–20)	22·2	21·6	22·6	<0·001	19·5	20·5	22·3	25·2	<0·001	19·3	23·8	22·5	<0·001
Underestimate (1–10)	36·5	34·8	38·1		32·5	37·1	37·9	36·8		32·4	35·7	39·8	
Overestimate (21+)	12·8	13·8	11·8		12·1	12·9	12·0	13·6		11·2	12·9	13·6	
Don’t know	28·6	29·8	27·5		35·9	29·4	27·9	24·4		37·1	27·6	24·1	
Minutes of jogging													
Approx. correct (31–60)	33·9	31·9	35·6	<0·001	32·3	33·2	33·3	36·0	<0·001	25·0	35·3	38·1	<0·001
Underestimate (1–30)	9·6	11·6	7·8		10·5	10·4	9·0	8·9		9·0	9·5	10·0	
Overestimate (61+)	17·7	15·0	20·1		13·9	17·5	17·8	19·8		15·9	17·4	19·0	
Don’t know	38·9	41·5	36·5		43·4	38·9	39·9	35·3		50·1	37·8	32·9	
Total calories (in calories)													
Approx. correct (201–300)	3·1	2·2	3·8	<0·001	2·2	3·2	2·5	3·9	0·008	1·6	2·8	4·2	<0·001
Underestimate (0–200)	6·7	5·5	7·6		5·6	6·2	7·3	7·2		5·0	7·0	7·6	
Overestimate (301+)	8·1	7·0	9·1		7·0	7·5	8·4	9·2		6·5	7·0	10·1	
Don’t know	82·2	85·4	79·5		85·3	83·0	81·8	79·8		87·0	83·3	78·1	

Approx., approximately.

Note. *P*-values are the result of chi-square tests.

In general, knowledge of the health effects tooth decay, weight gain/obesity, diabetes and heart disease as a consequence of soda consumption was greater among those from areas of least disadvantage, older adolescent age groups, and females (see Table 2; *P* < 0·001). Knowledge of cancer as a health effect was greater among those from areas of least disadvantage (*P* < 0·001). The belief that tooth decay, weight gain/obesity and diabetes are associated with diet soda consumption was higher among females, older age groups and those from least disadvantaged areas (*P* < 0·001). The belief that heart disease and cancer are a health effect of diet soda consumption was higher among older adolescent age groups and those from least disadvantaged areas (*P* < 0·001).

Almost two-thirds of adolescents (64·3 %) indicated that diet sodas were similar in healthiness to sodas, with 20·3 % reporting they were more healthy and 15·5 % reporting they were less healthy. The proportion indicating diet sodas were healthier than sodas was slightly lower among females than males (18·4 % and 22·4 %, respectively; *P* < 0·001), and appeared to decline slightly with increasing age (*P* < 0·001). The proportion reporting diet sodas were less healthy than soda appeared to increase with greater disadvantage (*P* < 0·001).

### Nutritional knowledge

Overall, 22·2 % of adolescents estimated the correct range for number of teaspoons of sugar in a 600-ml bottle of soda, with 36·5 % providing underestimates (see Table 2). Around one-third of adolescents (33·9 %) estimated in the correct range for minutes of jogging to offset the calories/kJs in a 600 ml bottle of soda, and very few adolescents (3·1 %) reported calories in the correct range, with most (82·2 %) indicating they did not know the amount of calories.

Variation in nutritional knowledge of soda was observed according to demographics (see Table 2). Correctly estimating the range for the number of teaspoons of sugar in a 600 ml bottle of soda was slightly higher among the 16–17-year-old age group (25·2 %) than younger age groups (19·5–22·3 %). Underestimating the number of teaspoons of sugar was slightly higher among females (38·1 %) than males (34·8 %; *P* < 0·001), and the proportion underestimating appeared to decrease as disadvantage increased (*P* < 0·001). Estimating in the correct range or overestimating minutes of jogging to eliminate sugar consumed from a 600 ml soda was slightly higher among females (35·6 % and 20·1 %, respectively) than males (31·9 % and 15·0 %, respectively; *P* < 0·001), and conversely, a slightly greater proportion of males underestimated (7·8 % and 11·6 %, respectively). The proportion reporting in the correct range for jogging was higher among the 16–17-year-old age group (36·0 %) compared to other age groups (32·3–33·3 %; *P* < 0·001), and estimates in the correct range appeared to increase as disadvantage decreased (see Table 2; *P* < 0·001). Proportions reporting ‘don’t know’ with respect to calories in a 600-ml soda were slightly higher among males (85·4 %) than females (79·5 %; *P* < 0·001), and showed a gradual decrease with increasing age (85·3 to 79·8 %; *P* < 0·001), and decreasing disadvantage (87·0 to 78·1 %; *P* < 0·001).

### Results of multilevel logistic regression analyses

Results of multilevel logistic regression analyses are available from Table 3. General knowledge (agreement) that consuming a 600 ml bottle of soda every day would likely lead to health problems in the future was not significantly associated with high soda consumption or high diet drink consumption in these models. With the exception of reporting cancer as a health effect of soda consumption, reporting specific health effects (diabetes, weight gain/obesity, heart disease and tooth decay) from soda consumption was significantly associated with lower odds of higher soda *and* diet drink consumption (see Table 3; *P* < 0·001). Those who indicated no illnesses were associated with soda consumption had significantly greater odds of both high soda (OR = 2·23, 95 % CI (1·79, 2·78), *P* < 0·001) *and* diet drink consumption (OR = 2·48, 95 % CI (1·90, 3·34), *P* < 0·001). The same pattern of associations was observed between beliefs regarding health effects of diet soda consumption and consumption of soda and diet drinks (see Table 3).

**Table 3:** Degree of association between knowledge, beliefs and consumption (soda and diet drinks), adjusted for school type, state/territory, sex, age and level of disadvantage

	Soda consumption – cups/week				Diet drink‡ consumption – cups/week			
	3 or less (ref)	4+	OR	95 % CI	3 or less (ref)	4+	OR	95 % CI
	%	%			%	%		
Likelihood of health problems†								
Not likely (ref)	12·0	16·6	1·00		12·3	17·4	1·00	
Likely	88·0	83·4	0·75	0·63, 0·90	87·7	82·6	0·73	0·57, 0·92
Reported illnesses ass. with soda consumption (ref: did not report)								
Diabetes	75·3	55·4	0·53	0·46, 0·61*	74·0	53·6	0·51	0·42, 0·62*
Weight gain	74·2	55·0	0·60	0·52, 0·69*	73·0	52·3	0·54	0·44, 0·65*
Heart disease	58·4	40·0	0·56	0·49, 0·65*	57·1	40·1	0·58	0·48, 0·70*
Tooth decay	78·3	58·0	0·54	0·46, 0·62*	77·0	56·0	0·50	0·41, 0·61*
Cancer	20·0	14·5	0·79	0·65, 0·95	19·6	14·8	0·82	0·64, 1·06
Unsure	11·5	16·6	1·21	1·00, 1·46	12·1	13·5	0·94	0·72, 1·22
None	4·3	14·4	2·23	1·79, 2·78*	4·8	16·8	2·48	1·90, 3·24*
Reported illnesses ass. with diet soda consumption (ref: did not report)								
Diabetes (Type II)	48·1	31·5	0·60	0·52, 0·69*	47·3	25·9	0·45	0·36, 0·55*
Weight gain	48·6	30·1	0·57	0·49, 0·66*	47·5	27·4	0·50	0·41, 0·61*
Heart disease	41·7	24·8	0·54	0·46, 0·63*	40·6	22·6	0·48	0·39, 0·60*
Tooth decay	58·6	37·3	0·52	0·46, 0·60*	57·1	37·0	0·53	0·44, 0·65*
Cancer	17·5	11·9	0·74	0·60, 0·90	17·1	11·5	0·71	0·54, 0·94
Unsure	29·7	36·5	1·26	1·09, 1·45	30·5	32·0	1·01	0·84, 1·23
None	5·2	16·1	2·16	1·75, 2·66*	5·9	17·2	2·12	1·64, 2·76*
Healthiness of diet sodas compared to sodas								
Same (ref)	65·6	55·1	1·00		65·3	48·9	1·00	
More healthy	19·6	25·0	1·43	1·21, 1·68*	19·3	34·6	2·31	1·88, 2·83*
Less healthy	14·8	20·0	1·40	1·17, 1·67*	15·4	16·5	1·27	0·98, 1·64
Teaspoons sugar in 600 ml bottle of soda								
Approx. correct (11–20) (ref)	23·3	14·3	1·00		22·6	15·2	1·00	
Underestimate (1–10)	37·3	31·3	1·34	1·09, 1·65	37·0	29·3	1·16	0·88, 1·54
Overestimate (21+)	12·7	12·8	1·51	1·18, 1·95	12·5	16·3	1·79	1·30, 2·46*
Don’t know	26·6	41·7	2·06	1·68, 2·52*	27·9	39·3	1·72	1·31, 2·27*
Minutes of jogging to work off								
Approx. correct (31–60) (ref)	35·6	22·4	1·00		34·8	20·2	1·00	
Underestimate (1–30)	9·4	11·1	1·62	1·27, 2·06*	9·5	11·1	1·77	1·27, 2·47
Overestimate (61+)	18·8	9·9	0·84	0·66, 1·06	18·0	12·4	1·20	0·87, 1·64
Don’t know	36·3	56·6	1·92	1·62, 2·26*	37·7	56·4	2·07	1·64, 2·62*
Total calories								
Approx. correct (201–300) (ref)	3·4	0·9	1·00		3·1	1·9	1·00	
Underestimate (0–200)	7·0	4·6	2·09	0·99, 4·45	6·7	6·6	1·32	0·61, 2·87
Overestimate (301+)	8·6	4·8	1·89	0·89, 3·98	8·3	5·3	0·91	0·41, 2·02
Don’t know	81·0	89·8	2·89	1·45, 5·74	81·9	86·2	1·17	0·58, 2·33

ref, reference category; approx., approximately.

* *P* < 0·001.

† Likelihood would have health problems from daily 600 ml soda consumption.

‡ Diet varieties of soft drink (soda), flavoured mineral waters, energy drinks, sports drinks and cordial.

The belief that diet soda was more healthy than regular soda was significantly associated with great odds of both high soda consumption (OR = 1·43, 95 % CI (1·21, 1·68), *P* < 0·001) and diet drink consumption (OR = 2·31, 95 % CI (1·88, 2·83), *P* < 0·001). The belief that diet soda was less healthy than regular soda was associated only with high soda consumption (OR = 1·40, 95 % CI (1·17, 1·67)).

Regarding the number of teaspoons of sugar in a 600 ml bottle of soda, compared to those estimating within the correct range, those who responded they did not know were higher consumers of soda (OR = 2·06, 95 % CI (1·68, 2·52), *P* < 0·001) *and* diet drinks (OR = 1·72, 95 % CI (1·31, 2·27), *P* < 0·001). Those who overestimated were also higher consumers of diet drinks (OR = 1·79, 95 % CI (1·30, 2·46), *P* < 0·001). Compared to those providing approximately correct estimates of jogging to work off a 600 ml bottle of soda, those who underestimated had significantly greater odds of being high soda consumers (OR = 1·62, 95 % CI (1·27, 2·06), *P* < 0·001), and those indicating they did not know had significantly greater odds of being high soda (OR = 1·92, 95 % CI (1·62, 2·26), *P* < 0·001) and diet drink consumers (OR = 2·07, 95 % CI (1·64, 2·62), *P* < 0·001). Estimation of calories in a 600 ml bottle of soda was not associated with consumption of either beverage.

## Discussion

This study aimed to explore knowledge and beliefs relating to the health effects of soda and diet soda consumption in the Australian adolescent population, and the degree to which knowledge of soda and beliefs regarding diet soda vary according to sex, socio-economic status, age and consumption of these beverages. The results showed that general knowledge of health risks of soda consumption was high (87 %), consistent with a previous study of adolescents^(26)^, and did not vary significantly by demographics, which indicates a near universal understanding among the demographic groups assessed that there is a general health risk associated with regular consumption of soda. Levels of knowledge of the specific health effects associated with soda consumption were relatively lower than general knowledge, ranging from 56 % to 76 % for diabetes, weight gain, tooth decay and heart disease, similar to a previous study of adolescents^(26)^. Reporting cancer as a health risk was relatively lower (19 %), indicating this disease is not as commonly perceived to be a risk of consuming sodas, similar to results of studies of adults^(30)^.

Beliefs that the same health effects are a result of diet soda consumption followed a similar pattern, though were somewhat lower such that they ranged from low to moderate (39–56 %) for diabetes, weight gain, tooth decay and heart disease, but were equivalent for cancer (17 %). Two-thirds of adolescents indicated they believed sodas and diet sodas were about the same when it came to ‘healthiness’. Adolescents may acknowledge that while both diet and regular sodas are not healthy per se, consuming sodas results in a greater risk of the health effects assessed in this study compared to diet soda. Adolescents may also consider consuming diet sodas to be associated with different health risks than those assessed in this study. Notably, around one-third indicated they were unsure of health effects associated with diet soda consumption, compared to 12 % that were unsure for soda.

In this study, adolescents had relatively lower nutritional knowledge of soda (nutritional composition, jogging required to eliminate sugar consumed) than general knowledge of the health risks and health effects of soda consumption. These results are similar to other studies that have also found higher general knowledge (e.g. general health risks, such as weight gain/obesity or overall knowledge a drink is sugary), and lower levels of more specific aspects of knowledge (e.g. sugar content, caloric composition, exercise equivalent)^(15,26,28,30–33)^. It is also consistent with qualitative findings among adults, that people have an overall sense that SSB are associated with health risks, but level of understanding of risk and actual physical implications beyond this initial sense is limited^(47)^.

Consumption of soda and diet drinks did not vary with general knowledge of future health risk with daily consumption of soda. This is likely due to adolescents’ perceptions of the distal nature of the health risk to themselves. This is similarly found in the area of tobacco among young adults, where long-term health risks of smoking have been found to be less compelling than short-term consequences^(48,49)^. However, soda consumption was very much in line with their knowledge of the specific health effects or diseases associated with soda consumption and their nutritional knowledge of soda (teaspoons of sugar or amount of jogging required for 600 ml soda). Having higher knowledge of the specific health effects of soda consumption and nutritional knowledge was significantly associated with lower soda consumption, even after adjustment for sex, age and socio-economic disadvantage. Conversely, reporting no health effects of soda consumption was significantly associated with higher consumption. Overall, this pattern of results was similar to findings of previous studies of adolescents that indicate specific aspects of knowledge tend to be significantly associated with consumption rather than more general knowledge^(24,25,27)^.

Taken together, the results indicate the potential for increasing several aspects of knowledge among adolescents; raising general awareness that daily soda consumption leads to health problems later in life, raising awareness of specific health effects associated with consumption and raising awareness of nutritional aspects of sodas (e.g. teaspoons of sugar, exercise equivalents). While higher soda consumers were less knowledgeable, the extent to which fulfilling these knowledge gaps would facilitate reduced soda consumption would need to be further evaluated and tested.

We identified several demographic subgroups which may assist in determining adolescents who would benefit most from intervention to increase knowledge of the health effects of soda consumption. While general knowledge of health risk did not vary by demographics (as previously described), knowledge of specific health effects and nutritional knowledge (calories, sugar content, exercise equivalent) did. Generally, slightly lower levels of knowledge were reported by males, younger adolescents and those from areas of greater disadvantage. An exception was that underestimating the sugar content of a 600 ml soda increased with age. This is particularly concerning given older adolescents may have more autonomy in purchasing and consuming these drinks. These associations are somewhat similar to those found among adolescents in two previous studies^(25,26)^. The gender difference is also reflective of demographic differences found in adolescents’ consumption behaviour in a 2012–2013 national survey of Australian adolescents^(41)^, perhaps suggesting increasing knowledge may reduce consumption and this demographic disparity between males and females. Associations between demographics with knowledge observed among adolescents in this study are also somewhat evident in adulthood, that is, among adults, knowledge has also varied by sex and level of education^(30)^. This is a novel finding and may indicate demographic disparities in knowledge commence when young but continue into adulthood.

To the best knowledge of the authors, this is the first study to explore beliefs regarding health risks of consuming diet sodas among adolescents and how these beliefs vary by demographic characteristics and level of diet drink consumption. Largely, demographic associations with beliefs regarding the health effects of consuming diet sodas were consistent with that found with sodas: lower health risks perceived among males, younger adolescents and those from areas of greater socio-economic disadvantage. A novel finding was that knowledge of soda health effects was significantly associated with diet drink consumption, in the same general pattern as that found with soda consumption across most knowledge indicators. For example, those who perceived specific health effects associated with soda consumption (such as diabetes) were also less likely to be high consumers of diet drinks. Notably, there were some associations that were stronger with diet drink consumption, although causality cannot be assumed due to the cross-sectional design of this study. Diet drink consumption was higher (over twice the odds) among those who considered diet soda to be healthier than soda, but it remains unknown whether health beliefs influenced, or were the consequence of, consuming diet drinks. Diet drink consumption was also higher among those with lower knowledge of the amount of jogging to eliminate energy consumed from sodas (underestimate or did not know), and the amount of sugar in sodas (overestimate or did not know). The results may indicate that some adolescents who are trying to avoid overconsumption of sugar may consume more diet drinks, or that their lack of knowledge is because they are less familiar with regular sodas. These results are interesting in light of substitution effects observed in some studies where consumption of ‘lite’ and no sugar beverages increased when strategies were introduced to reduce SSB consumption^(11)^. The findings of this study provide preliminary insight into how beliefs of beverages may relate to consumption. More research is required to determine the effects of educating consumers regarding sugar content of sodas on consumption of both soda and diet drinks. Results may also point to an overall higher level of nutritional knowledge/health literacy among low consumers of both sodas and diet drinks.

Limitations of this study include the cross-sectional design (no causality between knowledge and consumption can be inferred), and issues relating to the self-report nature of the survey rather than measurement of consumption via structured food recall interviews, and the associated potential risk for response biases. The knowledge measures utilised in this survey were all based on existing measures, however, would be strengthened with validation. Knowledge measures were focused on sodas, although it is noted that sugar and nutritional content can vary according to SSB type. It was not feasible to assess knowledge of nutritional content of the many types of SSB available within this study. Some questions of the survey were asked in reference to different volume sizes (e.g. 250 ml or 600 ml). Furthermore, while beliefs were asked about diet soda, consumption was assessed in relation to diet drinks. While ideally, volumes and drink types would be kept consistent across questions, this was due to different origins and purposes of questions. Specifically, consumption questions are important monitoring questions for the NaSSDA survey, and as such are kept historically consistent. Overall, this study presents data from a large sample of adolescents across Australia and explores associations that can inform future interventions and research.

## Conclusion

The knowledge gaps identified among adolescents in this study are informative for public health interventions, demonstrating both the need for intervention and aspects of knowledge that could be targeted to assist in reducing consumption. This study also identified specific demographic subgroups who may benefit most from intervention. Addressing these knowledge gaps will assist adolescents to make informed choices and likely encourage reduced consumption in some adolescents in the short term, and it will provide others with a greater understanding of potential harms, which they can draw upon as they become adults. In the context of inconclusive evidence on the health effects of ASB, there is a need to monitor the extent to which adolescents may consider diet drinks as an alternative beverage when reducing SSB consumption in response to public health interventions.
